# 44191 As Food Insecurity Worsens During COVID-19, Negative Mental Health Impact on Community Members Increases

**DOI:** 10.1017/cts.2021.768

**Published:** 2021-03-30

**Authors:** Catherine Striley, Piyush Chaudhari, Deepthi Varma, Linda Cottler

**Affiliations:** University of Florida

## Abstract

ABSTRACT IMPACT: For community engagement to be impactful and reduce health inequity, it needs to address timely needs in the community, including COVID-19 impacts. Here, we describe how pre- and post-COVID-19 food insecurity worsened mental health among community members served by HealthStreet University of Florida community engagement program. OBJECTIVES/GOALS: COVID-19 impacts the economic vitality and the mental health of communities; research and engagement activities must consider the context in which we are practicing and the needs of our community members. METHODS/STUDY POPULATION: HealthStreet, the University of Florida community engagement program, sends Community Health Workers (CHWs) where people congregate to assess social determinants of health and medical histories, used to make referrals to services and research opportunities. CHWs conducted follow-up COVID-19 assessments measuring perceived stress, loneliness, depression, anxiety, binge drinking, and opioid use, as well as high blood pressure and food insecurity. Here, we consider mental health outcomes among 1,300 adults who reported being food insecure either at some time in the past 12 months at baseline, or at the COVID-19 follow-up assessment, and completed both. Chi-Square Test was used to determine p-values. RESULTS/ANTICIPATED RESULTS: Overall, at the COVID-19 follow-up assessment, 37.1% (of 1,300) were still food insecure during COVID-19 (same), 20.3% (had become food insecure during COVID-19 (worse) and 42.6% were no longer food insecure (better). Those who were no longer food insecure were more likely to report less stress, while those still food insecure were more likely to report the highest stress and loneliness (p<0.0001), while the worse off group was in the middle. Those who stayed food insecure were most likely to report depression and anxiety, and also high blood pressure and using opioids (p<.05) compared to those getting worse or better. Binge drinking behavior was not significantly different across groups. DISCUSSION/SIGNIFICANCE OF FINDINGS: Community engagement activities across CTSIs must be sensitive to the needs of their communities. HealthStreet findings show that new and continuing food insecurity negatively influence mental health problems, pointing to the need for engagement to address multiple problems.

